# Psychological Quarterly Retrospect

**Published:** 1858-01-01

**Authors:** 


### *Psychological Quarterly Retrospect.*

THAT instant intelligent rapidity of correspondence with far-distant regions, which in popular mythology is a vain imagination, has become in our own days an accomplished fact. The most rapid flights of the "dainty Ariel" have been for some time emulated on the land, and, indeed, slightly also at sea; but now science has outvied that most exquisite spirit, in the passage even of the very ocean in which lie the "still vexed Bermoothes," and we may hopefully look forward to a period (it may be in our own life-time) when the boast of Puck—

> "I'll put a girdle round the earth
> In forty minutes"—

shall be effected by more legitimate and trustworthy, although less immaterial agency.

A little before sunrise on the 5th of August, the American termination of the Atlantic telegraph cable was landed in the Bull's Arm inlet of Trinity Bay, Newfoundland, and the deflection of the magnetic needles, attached to the termination of the cable immediately afterwards, showed that a communication had been established between the Old and New World. Notwithstanding that we have become familiarized with the working of the electric telegraph over wide-extended districts of the country, and even across narrow arms of the sea, the successful extension of upwards of two thousand miles of telegraphic cable, and the completion of an electric communication between two continents separated by a vast ocean, are achievements the importance of which, from their unparalleled character, it is difficult as yet rightly to estimate. The scene which occurred on the American shore, when the bond was completed between the two worlds, fitted well, in its solemn gravity, the great enterprise that had been accomplished. An eye-witness thus describes the landing of the cable:—

"At five o'clock, a.m., boats were lowered, and, accompanied by nearly all the officers and a great number of the crews of the ships, the cable was safely landed. By six o'clock the cable was in the telegraph station, and was no sooner connected with the instruments than a current of electricity was received from the *Agamemnon's* end. The landing had been performed without any cheers; officers and men fell in, in two rows, with the cable between them—the chiefs of the expedition in the van; it was thus dragged, or rather carried, to the station. Men now looked at one another as if they could scarcely realize the fact of the great work being accomplished. On every face the most intense relief and delight was depicted, and all felt it was now their duty to offer up their tribute of prayer and praise to that Supreme Power who had hitherto guided and blessed their efforts. All being assembled in front of the station, Captain Hudson" (taking up a position, according to another writer, "on a pile of boards, the officers and men standing round amid shavings, stumps of trees, pieces of broken furniture, sheets of copper, telegraph batteries, little mounds of lime and mortar, branches of trees, huge boulders, and a long catalogue of other things equally incongruous,") "delivered a short address, impressing on his hearers that the glory belonged not unto them, but unto Him who rules the raging of the sea, and holds the waters in the hollow of His hand. Prayers and psalms of thanksgiving were then read, after which all returned to the beach. Three such cheers now burst forth as none but English and American sailors can give; the hills and woods around echoed again and again, the crews of the ships joining in. The *Gorgon* fired a royal salute of twenty-one guns, with American and English ensigns at her mastheads."

Little needs adding to this simple yet grand picture of what will probably prove to be the most important event of the present period. It would be idle to speculate upon the political effects which may result from the junction of this country with America. That junction must lead to a closer union in the commercial and social intercourse of the two countries; and we doubt not that the closer these bonds are drawn, in proportion also the political union will become firmer.*

While science has, however, been putting to shame one of the most familiar attributes of the mythical personages who figure in our folk-lore, and leaving immeasurably in the rear the vapid vaticinations of modern clairvoyants, the old leaven of popular mythology has not failed to crop out from time to time in different parts of the country. The *Times* of September 2nd records the following instance of belief in witchcraft:—

Worship Street.—Sarah Macdonald, a middle-aged woman, living in Cudworth Street, Bethnal Green, was charged before Mr. D'Eyncourt with obtaining various sums of money under the following extraordinary circumstances:—

Mrs. Mary Anne Gable, a lady-like woman, dressed in mourning, and whose wan and anxious features plainly showed much mental and personal suffering, stated,—My husband is a coppersmith in Russell Street, Stepney, and having had a deal of trouble and illness lately, I heard and was convinced that a spell had been put upon me, and on the 23rd of last July I determined to go to the house of this woman, and did so.

Mr. D'Eyncourt.—But why did you go to her?

Witness.—Why, sir, she lays out the cards, and, indeed, is very clever with them. I had heard that she was clever in that way before, but I had not found that she had the power of relieving persons from torment by burning powders till then.

* The recent cessation of electric communication with Newfoundland from a defect, as yet inexplicable, in the Atlantic Telegraphic Cable is discouraging, and a free transmission of messages may be deferred for some time longer, if, indeed, it does not prove that the present cable is permanently injured. The thorough practicability of an electric communication between America and England is, however, now fully demonstrated, and it remains to be seen whether or not the mechanical difficulties can be overcome without involving a ruinous expense.

Mr. D'Eyncourt.—Why, what was the matter with you?

Witness.—Why, sir, I had such frightful pains, all cutting, shooting, pricking, and darting through my head and body.

Mr. D'Eyncourt.—Why did you not go to some medical gentleman about the pains you speak of?

Witness.—I did, sir; I was under Dr. Ramsbotham, and I asked him if he did not think they were caused by a spell; and he said, no, he thought not. He gave me some medicine, and for some time I got better; but I afterwards became worse from the pricking, and then, as I told you, I went to Mrs. Macdonald. She said, "You appear very ill," and I told her that I was. We had some conversation about it, and she then told me that a person was doing me an injury, and said, "If you have some of my powders, they will relieve you, but they are sixpence apiece." I told her, of course, that I did not mind that, and she therefore burned ten of them.

Mr. D'Eyncourt.—Then, she did not give them to you to take home?

Witness.—Oh, no, of course not. She put them into the fire before my face, and they all crackled, blazed, burned, and bounced.

Mr. D'Eyncourt.—What object did she say they were intended to effect?

Witness.—Oh, she said they would torment the person who was injuring me.

Mr. Safford, the clerk.—And did you feel any better from the powders being administered in that way?

Witness.—Oh, yes, I did feel better; but, mind, I don't believe that it lies so much in the powders as in the words she uses. I think it's what she says when she burns them that does you good.

Mr. D'Eyncourt.—Indeed! then what were the words she said?

Witness.—Oh, I don't know; she took care that I should not hear those, or of course I should be as wise as herself. She did not even mutter them.

Mr. Safford.—Then there was no incantation?

Witness.—Why, no.

Mr. D'Eyncourt.—Well, then, have you brought her here because you felt better, or what?

Witness.—Oh, no; I feel worse again—worse every day. I only felt better the first time I went. The fact is, that I have a relative who is coming into a large property, and she wants to get rid of me; so she goes to Mrs. Macdonald and has powders the same as I do, and of course they torment me whenever Mrs. Macdonald burns them for her. She knows that's true, too, for when I went another time she told me that the knowledge got made her so ill that she had been obliged to leave her husband's side in the middle of the night and burn a powder to get rid of the torments that she herself suffered from.

Mr. Safford.—And how often did you go to her?

Witness.—Oh, seven or eight times, I should think.

Mr. D'Eyncourt.—Did you ever tell your husband about these visits?

Witness.—No, certainly not; I did not want to tell him my troubles; he has quite enough of his own, I dare say; at least, if they are anything like mine.

Mr. D'Eyncourt.—And who told you so much about witchcraft?

Witness.—Well, sir, we all know that there is such a thing, of course.

Mr. D'Eyncourt.—Pray, where were you brought up?

Witness.—In the parish of Bethnal Green, sir; my father was a timber merchant, and had large property.

Horton, the officer.—The complainant's daughter is also present, sir.

Mr. D'Eyncourt.—Then let her stand forward.

A pretty buxom girl of 18 here got into the witness-box, and on being sworn said,—My name is Eliza Gable. I have been to Mrs. Macdonald's house several times, both with my mother and alone. When I first went there she said that a dark woman was doing us a great injury, but that she would put a stop to it. She described the dark woman, and she corresponded exactly with the relation we suspected. I have given her sums of money at different times, sometimes two shillings, sometimes one, and sometimes only sixpence.

Mr. D'Eyncourt.—Why, you look very well; what is it that ails you?

Witness.—Oh, I have suffered very much from her spells; I have very bad symptoms, I can't rest or sleep, and I feel as though I could fly out of the place. I believe that she is a witch, and has got the power of making spells.

Mr. D'Eyncourt.—And what did she do for you?

Witness.—Oh, why, she burned the powders, but they didn't do me much good. I believe that she can assist us if she likes, but that she wont.

Mr. D'Eyncourt (to the officer).—Have you any knowledge as to these complainants being respectable persons?

Horton.—Oh, yes; very, sir.

Mr. D'Eyncourt.—Are there many persons in Bethnal Green who believe in the prisoner's powers?

Prisoner.—They believe that I can bewitch the dead, but I can't.

Horton.—There are a great number, sir, who believe this, and I believe that the prisoner bribes several people to circulate the report. I apprehended the prisoner in her own house. I searched the place, and found these cards (about a pack in quantity, but of various colours and marked backs), and I also found the three powders I now produce.

Prisoner.—The children played with the cards, nothing more, and as to the powders, they are only salt. (The powders were opened, examined, and tasted, and seemed to be only what the prisoner said they were, common salt.) Mrs. Gable came to me and complained, but I told her from the first that I could not help her, and that she had better go elsewhere.

Mrs. Gable.—Yes, you told me so at last, because you found that you got more money from the other party than you did from me.

Prisoner.—She declared that she would sell her gown off her back rather than go without a powder. Even this very morning the daughter came to my house, threw a shilling down on the table, and said her mother had sent it for my great goodness to her. I told her that I had no power to help her mother, whose case really was a very bad one.

Mr. D'Eyncourt.—Well, I am truly surprised that a mother and daughter, who are not only respectable, but in other respects reasonable people, should be so very foolish.

Mrs. Gable.—Yes, I was indeed foolish to part with my money before she made the powders do me any good. I can't get any sleep.

Mr. D'Eyncourt.—How such simple notions can be entertained I cannot possibly conceive. There is no doubt, however, that the prisoner has been reaping a rich harvest from this kind of weakness, which I must put a stop to, and, though I shall now remand her, if upon inquiry what I strongly suspect should prove to be the fact, I shall send her to the House of Correction.

A lingering belief in popular superstition exists to a much wider extent among the population of this country than is commonly supposed. This belief is not only manifested from time to time by instances similar to that recorded above, but its influence is also witnessed, and that more extensively, in wide-spread superstitious delusions which ever and anon prevail. "Table-turning" and "spirit-rapping," regarded as results of supernatural agency, are familiar examples of delusions in which this lingering faith in common superstition is shown. "Table-turning" would appear to have been absorbed in spirit-rapping, and although this latter delusion is not manifesting, at present, much vitality in

England, it thrives with tolerable vigour in some parts of the Continent and in America. A highly favoured "*medium*" in Frankfort has of late been in communication with the spirits of Cæsar, Cleopatra, Homer, and Hippocrates, and he has not only obtained a fac-simile of the handwriting of each spirit, but Hippocrates has also given him a prescription, by the aid of which an old lady on the Rhine has been cured of acute rheumatism! In America "spirit-rapping" has for some time had the rank of a religious belief, forming one of several professions of faith which have been developed in that country within a short period; which ignore Christianity altogether; and which exhibit some of the most singular psychological phenomena that have ever been witnessed in the history of a people. The leaven of superstition which lies at the bottom of "spirit-rapping" determines those scions of the New World who have faith in it, to kick aside, with the ready restiveness of the political dogmas holding sway over them, the trammels of an Old-World faith; but the same leaven of superstition in the Old World is consistently had recourse to in order to strengthen the bonds of Christianity! We have scarcely recovered from the vision of "Our Lady of Salette," when we are threatened with another official visit of the Virgin in France. But let a correspondent of the *Times* (August 26th) tell the tale:—

"Well may the Parisian be proud of his metropolis; she stands alone, and need fear no rival.

"Yet with all the undoubted evidences of intelligence and enlightenment around, there is still a strange mixture of irreligion and superstition, which cannot be too deeply deplored. On Sunday, the 15th inst., the day of the Fêtes Napoleon, sparse indeed was the attendance at the various churches, while thirty-four theatres were crammed to repletion. At the present moment, a subject which in England would at once be scouted by rational men is here seriously discussed, and is implicitly believed in by millions. It is a miracle, or rather a series of miracles, which are reported to have been going on for some time in a grotto near Lourdes, not very far from Pau, and in the diocese of Tarbes. So much noise has the affair made in the south of France that the diocesan has formally appointed a commission to inquire into the circumstances.

"The story, it will be seen, is little more than a repetition of that of Notre Dame de Salette and the little cow-boys, but the following is the statement quoted from a recent ordinance of the bishop:—

"'Bernadette Soubirons, a young girl of Lourdes, thirteen years of age, has been favoured with visions in the grotto of Massavielle, at the east of the town. The immaculate Virgin has appeared to her; a fountain has sprung up (*surgi*) on the spot; the water of this fountain, applied either internally or externally, has effected many cures; these cures are reputed miraculous; people from our diocese and elsewhere have come in crowds, and are still thronging to the spot, requesting to be favoured with this water for the cure of various maladies. The attention of the civil power has been called to the matter, and on all sides, since the month of March last, the ecclesiastical authorities have been applied to for an explanation of these improvised pilgrimages.'

"The bishop proceeds to say that for his part he hardly thought it ripe for inquiry, but that his judgment has been overruled. There are, he adds, three classes of persons who appeal to his decision—those who look upon the whole thing as a cunningly devised fable and juggle; but, in the opinion of this worthy man, to deny the possibility of these supernatural occurrences would be to adopt the teachings of a superannuated school, and to revert to the infidel philosophy of the last century. Another class prudently suspend their judgment until competent authority has given its decision, which they await with anxious expectation. The third denomination includes those whose conviction of the genuineness of the apparition is already formed, and who, accordingly, hope for an announcement from the Church favourable to their pious sentiments.

"'For the purpose, therefore,' continues the prelate, 'of enlightening the minds of so many thousands of the faithful, we invoke the aid of Heaven to explain facts of the highest interest to the faithful aforesaid—*to the worship of Mary* (*la culte de Marie*), and to religion itself, and we hereby appoint a commission to inquire into all the circumstances.'

"The document is rather prolix, comprising eight 'articles,' the last directing the commission to commence its labours forthwith, and to meet as often as may be deemed necessary. It bears the signature of 'Bertrand, Sre., Bishop of Tarbes,' and the date of the 28th of July last. The commission, consisting of the deacons of the diocese, M. Nogaro (*chanoine-archiprêtre*) officiating as president, and MM. Tabariès and Soulé as vice-presidents, with a secretary and two vice-secretaries, and aided by scientific men, has already held several meetings, at which extraordinary revelations have been made by witnesses examined upon oath, and the result of their inquiry is looked for with an eagerness which would scarcely be credited in Protestant England. Meanwhile, pamphlets, comments from the press, and vehement discussions are rife, and the neighbouring provinces are in a ferment. How it will all end a few weeks will suffice to decide. Something, perhaps, may be hoped from the common sense of one at least of the clergy engaged on this inquiry, who, in reply to a deputation of his parishioners, requesting him to consecrate a tree of liberty for them—the thirtieth they had asked him to bless—is said to have replied that it was somewhat inconvenient to be continually called away from his other duties to perform this ceremony, more especially as the trees usually died; he therefore suggested that they should adjourn to the nearest nursery-ground, when he would bless every tree in it once for all, and they would thus have a stock of consecrated timber to draw upon at will."

It would be difficult to estimate whether the "spirit-rapping" of America or the saint-visions of France exhibit the most pitiable spectacle. Whatever intermediate agencies may come into play in the development of these popular delusions, *ignorance* is at the root of the matter, and it is this which is either worked upon, or out of which the delusion arises spontaneously. We may not so readily exhibit such gross instances of religious delusion in this country as are witnessed in America and France, but we must not lay too great unction to our souls on account of this. The taint of a delusion is at least sure to rest upon us. There is, we believe, a chapel of "Our Lady of Salette" in England. We have had a tolerably wide, although mild epidemic of spirit-rapping; but the outbreak of table-turning among us was of a serious character, and to one or two of our clergymen that epidemic was indebted for the gloss of its Satanic character.

Mormonism has a hold among us (and some of its moral peculiarities have recently been exhibited in the metropolitan police courts), and we do not want other-*isms* of very doubtful morality, and certainly deficient of any Christianity whatever.

The great centre of Mormonism in the Utah territory has, after a brief rebellion, been brought within the jurisdiction of the United States. The *Times*' correspondent in the Great Salt Lake City remarks, concerning the peace which has been concluded between the Mormons and the States, that "the Government has succeeded in effecting something which they call a peace, but which will prove to be a mere repression of the moral ulcer, to be followed at no distant day by a reaction far worse than the original disease." The same writer gives the following highly interesting particulars respecting the two principal leaders of the Mormons :—

"I called upon Brigham Young. I found him a well-presenced man of fifty-seven years of age, of medium height, of figure rather inclined to corpulency, with sandy complexion, and a vulgar sensual mouth. He was well, but plainly dressed, rather austere in manner, and evidently fully conscious of the necessity of maintaining a sort of royal dignity, becoming a prophet. I should judge him to be shrewd in worldly affairs, a good business manager, a judge of human nature, and capable of adapting it to his will. The cast of his mind, however, is evidently low and vulgar. While shrewd and cunning, quick and ready in the application of what powers of mind he possesses, the prophet is by no means a wise man nor profound; and in discussion with an ordinarily skilful opponent he fails utterly. Nevertheless, his power over the people is limitless. His nod is law; and the ignorant masses of his followers look upon him as almost a God. I had the pleasure of hearing him deliver a sermon on the Sabbath, in the course of which he quite satisfied me that I was not mistaken in my estimate of his mental calibre. His discourse was rambling and vulgar, although his manner was popular and forcible. He never rose to the dignity of an argument, but all his positions depended for success upon the blind acceptance of his own dicta. He referred to the army of the United States as ruffians, and then made a lame effort to cover up the blunder he had sense enough to perceive that he had perpetrated. He spoke of the President of the United States as 'an old dotard, whose friends *allow* that he ought to have been elected twenty-five years ago, when he had a little sense about him, if ever;' and in urging the 'sisters' not to hurry their husbands back to their homes, told them, if it made their heads ache to live in tents, to 'go out and get a chip to put on their heads.' This specimen is quite sufficient to satisfy and disgust every intelligent reader. Judge what must be the misery of an educated and refined proselyte to Mormonism, who comes here as to a heaven upon earth, and finds the prophet, the vicegerent of God, a man of such vulgarity as the above language marks him.

"But Brigham is a model of elegance and refinement compared with Heber C. Kimball, the next in the priesthood. He is only a few days older than Brigham, is tall, full formed, with short sandy hair and whiskers, florid complexion, and small, cunning, snake-like black eyes. No one knows with certainty how many wives Brigham has, but Heber pleads guilty to about forty, by whom he has only about fifty-eight living children, having lost half-a-dozen. His reputation as a husband and father is bad, and many are the secretly-whispered tales of his jealous cruelty to his wives, some of whom are younger than his first-born child. He is certainly the most vulgar and blasphemous wretch it has been my misfortune to meet. Excepting the use of the name of God, there is no form of blasphemy which is not familiar to his lips. He assured me that he loved his friends and not his enemies. Being rebuked for this sentiment by a Gentile bystander, he declared that he followed the Scripture, nevertheless, and prayed for his enemies. This sentiment elicited commendation, when Heber continued—'Yes, I pray they may all go to h—ll and be damned.' This, let me assure you, is a fair sample of the style of language employed by this second member of the priesthood, in the pulpit and out of it. Another illustration of his spirit, and I leave Brother Heber. He was asked if he would resent an insult by violence; and he responded, 'The Scriptures tell us that if smitten upon one cheek we must turn the other also. Well, I'll do that; but if a man smites me on the other cheek too, *let him look out for a — of a lick back!*'"

Unsatisfactory as the morality of Mormonism may be, it is fated to be outdone. An American correspondent of the *Times* (July 12th) writes as follows:—

"Persons dissatisfied with the rest of the world, and admirers of themselves, have just been gathered together under a canvas tent among the mountains of Vermont, with an explosion of gasconade ribaldry and lasciviousness that puts Brigham Young to the blush and Mormonism in the shade. Marriage is denounced as the cause of the slavery and degradation of woman, whereby she loses the control of her name, her person, her property, her labour, her affections, her children, and her freedom; and a pretty delegate from New York—representing what department in our Babel I cannot say—on 'the sunny side of 30,' with pretty curls, bewitching smile, sparkling eyes, and sweet elocution, held forth upon this text in a speech that fills a column and a-half in the morning journals. She treated her subject *con amore,* but in language that will hardly bear transcribing. Discontented minds of every sort were there represented, complaining,—some of the restraints of the marriage tie, some of the weight of the Church and religious institutions, some of the sin of slavery, some of the unjust distribution of property—all of the sinful mote in the eyes of offending friends and strangers, none of the beam that was blearing their own vision. Two or three times a-year there has to be just such a 'stampedo' of 'isms.' The public is amused or shocked, according to its fancy, and the spread of these peculiar tenets is effectually checked by the absurdity of the display."

A parallel to these socialistic doctrines may be found in France. The Paris correspondent of the *Standard* writes (June 28th):—

"The tribunal of correctional police of Lyons has at length pronounced its verdict in the affair of the secret society detected in that city many months ago. Thirteen individuals were arrested, but many of the suspected parties succeeded in gaining Switzerland and Savoy. Eleven of the accused were condemned to terms of imprisonment varying from one to two years, and to small fines; the remaining two were acquitted. One of the prisoners, named Bernard, while on his defence, indulged in the following abominable language:—'Yes, gentlemen, I am an atheist. I proclaim it aloud. Society will crumble, for it is organized in an abominable manner. Incest is not a crime. Were brothers permitted to marry their sisters, you, gentlemen of the bench, would have less occupation, for there would be a less number of assassins and thieves, consequently less crimes and offences.' This amiable specimen of the French Socialist concluded his defence thus: 'The best proof that I do not belong to any secret society is that I have given you my word of honour to that effect.' The scamp was sentenced to one year's imprisonment and 100f. fine."

It is a curious and at the same time most instructive psychological study, to witness the development of the popular delusions of our own time. We may discern the same essential principles at work as are seen in the delusions of remoter periods, but in addition we may mark the impress of the peculiar social or scientific tendencies of thought which more particularly characterize the present period. The popular delusions of our own day, indeed, grow from roots similar to those of the delusions which held sway in the mediæval and earlier ages, but they have a new guise, and one more befitted to the existing tone of thought. The disposition which still exists so widely to entertain the various social, religious, and pseudo-scientific delusions, which have cropped out from time to time during the last twenty years, provokes an inquiry into the *quality* of the education which is dealt out to the middle class; for we are inclined to attribute the persistence or origination of popular delusions more to the defective knowledge of this than of any other class of society. It has long been believed that the education of a very large proportion of the middle class is not of the good quality which might have been expected from the great efforts which have been made in recent years for the better instruction of all classes of society. The late Oxford examination of "Persons not Members of the University," has shown some of the deficiencies of middle-class education in a very strong light. In a leading article, the *Times* sums up the general result of this examination in the following language:—

"The first barrier was the preliminary examination, and it ran upon matters commonly supposed to be taught to a young gentleman by his governess and a village tradesman's son in the first-class of a national school, but seems to have proved fatal to half the rejected. The preliminary examination was in orthography, writing, analysis and parsing, arithmetic, geography, English history, and English composition. If some of these subjects were a little higher than the ordinary scope of a village school, at least the 'Commercial Academy' ought to teach them. However, it appears that though half would have been rejected anyhow, half would have got through, and many of them would have obtained honours but for this little difficulty. This is so remarkable a fact that it calls for the explanation of those who talk of the diffusion of knowledge and the success of the voluntary system. No doubt much may be said, and, indeed, much does occur, to throw light on this singular phenomenon, but we should like to know what is said. A candidate for this examination must have not only some degree of confidence, but also some credit with his friends. We assume that he is seldom such a one as his school would disown, and that his friends have looked kindly and hopefully on his attempt at university distinction. Yet he cannot write good English; he makes mistakes even in his spelling; he breaks down in the first four rules of arithmetic. If he were engaged as a steward, a butler, or a head-gardener, he would betray his ignorance on the first occasion he had to write a letter to his master or send in an account. He might use very fine words; he might have an immense horticultural vocabulary; he might even talk volubly and grandly; but put him down to pen, ink, and paper, and his grand words would be ill spelt, his constructions would defy syntax, his letter would jump from one person to another, and even his figures would be untrustworthy. There would be serious errors, of course in his own favour, for otherwise a poor man could not leave them undetected in his monthly or yearly accounts. If such a man were appointed to a school he might go on for a long time without detection, he might never be detected, and he would go on teaching bad spelling, bad grammar, confusion of thought and ideas, bad reasoning, and bad arithmetic all his days, till he had sent out into the world several hundred obstinate blockheads or conceited fools. So we cannot doubt that the application of the Oxford test has been very salutary in this instance; that it was wanted; that the result justifies the wisdom of the so-called Middle Class Examination; and that, as the examiners sharpen the test, which they promise to do, they will find the candidates better qualified to stand it. . . . . Looking, then, to the general results, the most remarkable, as it seems to us, is the fact that, when five-eighths could not pass the preliminary examination, more than two-thirds of the whole, above eight hundred, volunteered the examination in the 'Rudiments of Faith and Religion;' and of these a very small proportion failed to satisfy the Divinity Examiners, while the greater part are said to have passed creditably. In the middle class, therefore—the class which cannot afford an Oxford education, and is not too proud to seek its testimonials—there appears to be more religious than common information. The failure is in writing, grammar, arithmetic, English history, modern geography, and such matters supposed to be known by everybody one meets—*not* in doctrine or Bible history. This was to be expected, perhaps, from a body of candidates sent up from our village schools; but we were not quite prepared to expect it from any higher class. There appears to be some difficulty in the working of this part of the scheme—at least, the schoolmasters find one—and an alteration is expected. The honours have fallen very naturally to good grammar and proprietary schools, and there cannot be a more desirable result than that these schools, conducted by able and hardworking men, and under good management, should have this testimony to their merits. But the crop of honours has been shared by evening classes and institutes. That, too, will work well. There are, happily, many ways in which a man may improve himself. Indeed, where there is a will there is always a way. But one way is better than another, and it fairly belongs to such a body as Oxford to show the best way."

The results of the voluntary examination in the "Rules of Faith and Religion" are certainly somewhat surprising, although gratifying, and it is to be hoped that they will prove a "shadow of good things to come;" for it is only as the moral and religious standard of the middle class is raised, that we may hope for improvement in the morality of the lower classes.

We may not, in jotting down those items which indicate the psychological tendencies or phenomena of the quarter, discuss the causes of that defective education which prevails too extensively among the middle class; but the following anecdote, told by a recent tourist in Skye, shows well *one* cause which we believe operates very generally in the education of all classes of society:—

"One little incident we must mention, as illustrating education. Walking to church one Sunday in Skye, we were followed by a slip of a lad some ten or eleven years of age, who, on putting some questions to him, volunteered to name all the capitals in Europe, which he did with marvellous dexterity. From Europe he crossed to South America, and rattled out the names of the capitals with the accuracy of a calculating machine. From South America he started off to Asia, and finally brought up at Jeddo in Japan. We were rather sceptical as to the value of such acquirements, and, indeed, as to the reality of any information having been conveyed to the lad's mind by the formidable muster-roll of words that had been stuffed into his mouth. We therefore asked him, 'Can you tell us the name of the island you live in?' But, notwithstanding his lore, he had not learnt that he lived in the Isle of Skye. To make quite sure of the fact, we requested the captain of the steamer to repeat the question in Gaelic; but there was no Skye forthcoming. He knew the name of the parish, and of all the capitals in the world, but not of the island he lived in. There being a schoolmaster present accidentally, we thought the occasion too good to be lost, to show the worthlessness of word stuffing, and ventured another question. 'Now, my lad, you have told us the names of nearly all the capitals in the world; is a capital a man or a beast?' 'It's a beast,' said the boy, quite decisively. So much for words without understanding. In the next school inspection that boy will probably pass for a prodigy, and will figure in statistical reports as an example of what good education can do."—*Glasgow Commonwealth.*

"Avarice, after the description of Seint Augustine, is a likerousnesse in heart to have erthly thinges. Som other folk sayn, that avarice is for to purchase many erthly thinges, and nothing to yeve to hem that han nede. . . . . The difference betwene avarice and coveitise is this: coveitise is for to coveit swiche thinges as thou hast not; and avarice is to witholde and kepe swiche thinges as thou hast, without rightful nede." Here is a rare comment upon this paragraph of Chaucer's "Persones Tale":—

"Ye who listen with credulity to the whispers of vanity, and pursue with eagerness the phantom of a name, attend to the history of one richer than Rasselas—even to the history of one Peter Thellusson, late of the city of London, merchant. It is partly detailed in the columns of our this day's Law Report, but scarcely plainly enough to be understood without labour by non-legal minds.

"It is now sixty-two years since Peter Thellusson took stock of his worldly possessions, and found that he had 600,000*l.* in money, and land of the annual value of 4500*l.* Peter Thellusson had satisfied the ordinary ambition of an English *bourgeois*—he had founded a family. Peter Isaac, the son of his youth and the prop of his house, was heir to 35,000*l.* a-year in money and land, and might claim to be a born gentleman. Peers and peeresses might hereafter spring in intermediate succession from the loins of that denizen of a dingy little back parlour behind the Bank. The best men upon 'Change envied the rich and prosperous Peter Thellusson, who had no object of ambition unsatisfied. Peter was of a different mind; he had not nearly money enough. Let other men be satisfied to found one family; Peter was lucky enough to have three sons, and he would found three families. It was not that he loved his sons, or his sons' sons; but it was the hope and desire of this magnificently posthumous miser to associate his name in future generations with three colossal fortunes. If he did not love his sons, he did not hate them; he was simply indifferent to everything except to his one cherished object. Peter Thellusson took the very best legal advice, and made a will. He left a few trifling legacies, probably to show that no unnatural antipathy to his children tainted that will with mania.

But his great fortune was all conveyed to trustees It was to accumulate until every man, woman, and child of the offspring of Peter, and alive or begotten at the moment of Peter's death, should also be defunct. No one of the children or grandchildren who had ever looked Peter in the face, or trembled in his presence, or squalled at the sound of his harsh, hard voice, should ever be the richer for Peter's wealth. 'And the rich man also died.' Twelve months after making this will, and sixty-one years from the present time, Peter was gathered to his unknown fathers. The will was opened, and created sensations which vibrated through the land in widening circles. Our law books picture to us the blank disappointment of the then living relatives, the gentle cachinnations of a past generation of lawyers, and the gaping wonder of the general public. There were three sons and six grandsons of this malignant old merchant then alive—all destined to live the life of Tantalus; to see this great pagoda-tree growing up before them, yet never to pluck one unit of its fruit. The terms of the will enjoined, that when the last survivor of all the nine children and grandchildren should yield up his breath, then the charm was to end; the great mountain of accumulated wealth was to be divided into three portions, and one-third was to be given to each of the 'eldest male lineal descendants' of his three sons. Having thus done what he liked with his own, and excluded all his living progeny from all benefit, he ends with a whine to the Legislature worthy of Shylock appealing against mercy—he had earned his money with honesty and industry, and he hoped the Legislature would not alter his will. Of course, the first thing that followed was a Chancery suit of the fattest bulk. The common-sense view of the case would have been to set aside the will as the product of a diseased mind—a mind rendered morbid as to its disposing powers by dwelling upon an irrational object. But Lords Loughborough and Alvanley and Eldon, and judges of kindred sympathies, seem to have been led by their love of art to admire the skill with which the technicalities of our blessed real property law had been adapted to the object of this old trader. Perhaps, also, they saw something eminently sane and matter-of-fact in this good old sordid vice of accumulation, or were excited to admiration by seeing the meanest vice of man expanded into something like sublimity in its gigantesque proportions. The litigation went up to the House of Lords, and the will was confirmed. This affair naturally made a great noise. The Legislature took it up, and, although they would not set aside the will by an *ex post facto* law, they branded Peter Thellusson's memory with the imputation of 'vanity, illiberality, and folly;' and enacted by statute 39th and 40th of George III., cap. 98, that the power of devising property for the purpose of accumulation shall be restrained in general to twenty-one years after the death of the testator. Persons of an arithmetical and statistical turn of mind also occupied themselves with the matter, and with the aid of life-insurance tables and Cocker, they calculated that this fund, accumulating at compound interest, could not amount to less than nineteen millions at the moment of distribution, and would very probably reach the tremendous figure of thirty-two millions. But 'nothing is so false as facts, except figures.' The calculators had forgotten to take account of that unknown quantity which must, in practical matters, be represented, not by the letter '*x*,' but by the word 'litigation.' Contemporaneously with the Chancery suit to set aside the will there was a cross-suit to have the trusts of the will performed under the direction of the Court of Chancery. That suit is now sixty years old, and, although children and grandchildren are dead, the suit is as hale and lively as it was in their earliest youth. That suit was the true heir to Peter Thellusson, and it is still spending his money like a frolicsome young cornet. Necessarily, there were other suits. There were suits about post-testament acquisitions of real property, there were suits about advowsons, there were suits about other matters, so numerous that even equity lawyers, not stingy of their words, are fain to describe them as 'various.' The careful and improving management of the Court of Chancery has also exercised its influence upon this estate. The Yorkshire estates have participated in that excellent system, which has been so uniform in its action, that when we see a house all windowless and unpainted, tottering and decaying, we can predicate with a tone of undoubting conviction, 'That property is in Chancery.'

"The last survivor of the nine lives died in February, 1856, and four new bills were immediately filed. The property is now to be divided, not into thirds, but into moieties. There is, however, a question raised as to who is entitled. Who were the eldest male lineal descendants of old Peter Thellusson in February, 1856? There are two who are eldest in point of lineage, and two who are eldest in point of personal age. This point is still *sub judice*. It would not be very difficult to guess how it will be decided; but that is no matter of ours, nor would it have been a matter of the least interest to old Peter Thellusson. His object was to make the heap very large; he evidently cared not one lock of wool as to which of his descendants might be the possessors. The public interest in this long line of litigation is confined to its general aspect. Peter Thellusson's clever scheme has turned out a foolish failure. No single Thellusson will stalk over the land, overshadowing our dukes and crushing our barons by the magnitude of his territorial possessions. No thirty-two millions of money are expanded into broad acres, where men may travel and say—'Behold the conquests of the great Peter Thellusson.' Whether Lord Rendlesham and Charles Sabine Augustus Thellusson divide the estate as the eldest in lineage, or whether Thomas and Arthur take as eldest in years, we should equally desire to be able to call up old Peter Thellusson to see the division of his anticipated accumulations. The Court of Chancery has so clipped and pollarded his oak, that it is not much larger than when he left it. It would be fit punishment for that purse-proud, vain, cruel old man to see that he disinherited his own children only to fatten a generation of lawyers; that he was the dupe of his own subtlety, and that his name, instead of being associated with the foundation of a house of fabulous wealth, is only known in connexion with an abortive scheme of vulgar vanity."—*Times*, July 5.

We think with the *Times* that "the common-sense view of the case would have been to set aside the will, as the product of a diseased mind." The following recitals may be placed in the same category as the act of Peter Thellusson:

"A singular lawsuit, which has been pending for several years at Ferrara, has just been amicably settled. A nobleman, named Bonaccioli died some years ago, leaving a will by which he appointed his own soul as universal heir to his estates, representing a value of 5,000,000f. The charitable institutions of Ferrara laid claim to the property, while the brother of the deceased attacked the will on the ground of nullity. After long judicial debates an arrangement has at length been come to, by which the brother abandons his claims in consideration of certain moneys which Cardinal Casoni, the curator of the above establishments, engages to pay to him, and to the other relations of the deceased. It appears that this result has been obtained through the interposition of the pope; in any other country the will would have been declared null and void."

We read in *Galignani*:—

"Society in Vienna has been recently startled by the following strange act accomplished on his death-bed by a Baron Silberstein. He had threatened to disinherit his son in consequence of a family dispute; and on feeling his end approaching, he carried out his threat; he converted his fortune, amounting to 170,000 florins, into bank-notes, and burnt them."

Public attention has of late been directed very much to the subject of insanity, and the care of the insane. Several cases, in which the plea of insanity was raised, and which were calculated to excite general interest, have, during the past quarter, been heard in our law courts, and several commissions of lunacy have also been held.

Foremost in interest among the commissions was that on Mrs. Turner, not so much from any intrinsic merit in the case, as from the influence it had in giving rise to a popular outcry against private asylums; foremost in importance was the commission on Mr. Ruck. The commission held to inquire into the state of mind of Sir Henry Meux, M.P., is worthy of being noted from the length of time, nine days, which it occupied, and from the fact that the jury were not able to come to a conclusion from the evidence given upon the time when Sir Henry first became of unsound mind (the point submitted to them), it being admitted that he was imbecile at the time of the inquiry.

The commission appointed to inquire whether Mrs. Turner was a lunatic or not, was held at York in July. Mrs. Turner had been sent to the Acomb House Asylum, near York, in December, 1857. At that time she was undoubtedly lunatic, the most marked symptom being a suspicion that a conspiracy had been entered into, her husband being connected with it, to "murder" her by poison. The jury, consisting of twenty individuals, after the inquiry had extended over two days, returned a verdict (seven jurymen dissenting) to the effect that Mrs. Turner was then of sound mind. The case itself presented no points of particular interest, but during the inquiry it was ascertained, upon the evidence of the proprietor of Acomb House himself, that he had at times been guilty of using grossly offensive language to Mrs. Turner, and that he had also treated her occasionally most brutally. The conduct of the proprietor was shortly after made the subject of special investigation by the commissioners, and upon their recommendation the licence of the asylum was revoked.

The unjustifiable treatment which Mrs. Turner had received at Acomb House was made the subject of severe stricture by the press, and occasion was taken, at the same time, by several influential journals to make an attack upon private asylums generally; and subsequently an almost general onslaught was made by the press upon these establishments, the commission on Mr. Ruck contributing not a little to such a result.

Mr. Ruck is a gentleman whose usual residence is in Merionethshire. Seventeen years ago he had married a Welsh lady, and he had lived with her until the period when it was thought necessary to confine him in an asylum, having by her a family of six children, the youngest being at the time of the inquiry two years old. It was shown in the evidence given before the commission that, in 1856, he had given way to intemperance at intervals, and that occasionally he manifested extraordinary restlessness, going about at all hours of the night without apparent object, and sometimes causing himself to be driven over unfrequented tracts of country in the dead of the night. In 1857 he became subject to extravagant delusions respecting the virtue of his wife; he also entertained a belief that two illegitimate children born to him by a female residing in his own house had been murdered; and several times he manifested suspicions that the food given to him was poisoned. The delusions respecting his wife's fidelity increased upon him. Often he expressed them to her in the foulest language, accusing her of the vilest and most promiscuous prostitution; and in the latter part of the year, on the advice of Dr. Conolly, he was removed to Moorcroft House Asylum. It was admitted that the delusions which led to Mr. Ruck's confinement were present at the commencement of June last, but it was contended that they had been entirely removed since that period, in consequence of the inquiries set on foot, for Mr. Ruck's satisfaction, by his solicitor. Dr. Forbes Winslow gave evidence of the existence in May of the principal delusions under which Mr. Ruck suffered, and Dr. Conolly and Dr. Sutherland gave evidence of their existence at the commencement of June. These physicians had not had an opportunity of examining Mr. Ruck since June, but from the opinion they had formed of the character of his affection when last they had examined him, and from the evidence which had been since laid before them, they thought that there was not sufficient evidence to show that the delusions were removed, although they considered Mr. Ruck to be much improved. Moreover, they did not believe that intemperance was the sole cause of his delusions. Dr. Tuke, Dr. Seymour, Mr. Lawrence, Mr. Skey, Mr. E. Canton, Dr. Copeland, Mr. John Gay, and Dr. G. Johnson, all of whom had examined Mr. Ruck since June, and since proceedings relative to the commission had been commenced, gave evidence as to his then sanity, and all, except Mr. Lawrence, expressed the opinion that the delusions had been caused by intemperance. The question of treatment was raised during the inquiry, and the majority of the medical witnesses expressed opinions coinciding with that given by Dr. Forbes Winslow to the effect that it would perhaps have been better had the case been dealt with out of an asylum; and Mr. Lawrence thought it was still requisite, as a matter of prudence, that Mr. Ruck should for a short time be kept under skilled supervision. After the proceedings had lasted five days, the jury, consisting of eighteen members, returned a verdict to the effect that Mr. Ruck was of sound mind, and capable of attending to his affairs. Six of the jury dissented from the verdict.

Perhaps the most interesting part of Mr. Ruck's case was the allegation that the delusions had been removed in consequence of the inquiries made by his solicitor. The following is Mr. Ruck's statement with regard to this:—

"Mr. Ruck, the alleged lunatic, was next called, and, in answer to questions put by the Commissioner, said, 'I have been present during the whole of this inquiry. That is my misfortune, though it has not disturbed me very much. I have heard the whole of the evidence. I was in a confused and agitated state of mind when I entertained these suspicions about my wife. I was caught up and put into this asylum at Moorcroft without having had any opportunity of investigating the circumstances. I merely wished for inquiry, and as soon as I had been satisfied that there were no grounds for my suspicions I should have abandoned them. . . . . As I got well, the suspicions nearly vanished. I cannot say they were entirely removed from my mind, and I wished to ascertain if there were any grounds for them. I asked my solicitor, Mr. Wainwright, to investigate the facts. Though I was under those notions for a time, the facts have been now investigated to my satisfaction.'

"The Commissioner.—What means has your solicitor taken to investigate the facts?

"Mr. Ruck.—Ample means, I believe; and he took a very long journey to do so. He made the investigation at my request, and after he told me the result I said, 'That will do,' or something of that sort. I could not disabuse my mind before that investigation.

"The Commissioner. — That investigation having been made, you are satisfied?

"Mr. Ruck.—Perfectly satisfied. The investigation he made has satisfied me that my suspicions were purely imaginary.

"The Commissioner.—It would be satisfactory to the jury to know the facts on which you have come to that conclusion.

"Mr. Ruck.—I believe that the gentleman who travelled with us to Welshpool had not seen my wife in the previous part of the morning. That was one of my suspicions. I had also a suspicion that Mary Jones came and lay by my side at Welshpool. It turns out that Mary Jones was never at Welshpool at all at that time. Having felt those suspicions, I could not rest until I had satisfied myself that they were groundless. I was bound to investigate them. Another circumstance is that I made a mistake about the name of the gentleman who travelled with us in the mail to Welshpool. I find his name is Bailey, and not Peach, which I supposed it to be. It is possible, in the excitement under which I laboured, that I conceived many things true which were not. I can only say that I don't think anything more of them. I wished an investigation to be made at Reading when I was there, but I was prohibited from seeing any of my friends or writing to them. It was on the 14th of June last that Mr. Wainwright communicated to me the result of his investigation. I have not seen Mary Jones since I left her at my own house. The children are an unfortunate affair. I don't know where they are now. I have never seen the children I had by her.

"The Commissioner.—Do you think they are living?

"Mr. Ruck.—Well, I suppose they are. She refused to give me any information about them. She would never say whether they were alive or what she had done with them. She ought to have satisfied me about that. . . . . .

"The Commissioner.—Why did you not communicate to her (Mrs. Ruck) that your suspicions had been dispelled?

"Mr. Ruck.—Because I had then got notice of this commission, which I thought was a most cruel thing towards me. If she had not issued this commission, and had allowed me to institute this inquiry, everything would have been arranged satisfactorily. I have lived in this country all my life, and I could not understand that such a thing would have been permitted as the sending me to Moorcroft in the way in which I was sent there. I suspected my wife on insufficient grounds, and now that my suspicions are removed I am satisfied. There is no foundation whatever for my suspicions that she was criminal with other men. I made those charges under excitement arising from drink. I had been quiet up to within a week of my leaving Pantlludw, and able to transact any business. I went to amuse myself in shooting three or four days, and then the weather changed. I drove about the country to different places to amuse myself, and took drink, and that accounts partly for the state in which I was found. . . . .

"By the Commissioner.—He had for some years lived unhappily with his wife, and been in consequence very wretched. That was owing to her refusing his having intercourse with her. He desired to be separated from her, because he understood that she did not wish to live with him."—*Times*.

Mr. Ruck's case attracted great attention from the public, not only on account of the distinguished names of several of the medical men who gave evidence before the commission, but from the fact of the inquiry occurring at a time when a strong feeling existed against the management of private asylums; and one circumstance connected with the case—the careless mode in which a portion of the certificate consigning Mr. Ruck to the asylum had been filled up—seemed to justify the press in the course of argument it had, almost without exception, taken upon the question. We do not propose to enter here into any justification of or plea for private asylums; and Dr. Forbes Winslow has already commented on the unsatisfactory state of the law regarding medical certificates of lunacy.* We would, however, remark, that the supervision of private asylums by the Commissioners of Lunacy is of a much more effective character than that of public. For example, the occurrences at Acomb House led to an immediate revocation of the licence; but we are told, in the last Report of the Commissioners, that they had had reason to complain of the foul condition and defective general treatment of the patients, and also of one or more acts of excessive cruelty, at the public asylum in Haverfordwest; yet notwithstanding the remonstrances of the Commissioners at four different visits, at intervals of several months, no improvement was made in the asylum, and at length they were compelled to submit the matter to the Secretary of State. Now, the *Times* says, "We are fully prepared to acknowledge that there are private asylums conducted by persons of high character, in which every comfort and care that can be desired are afforded to persons labouring under mental disease; others there are, again, in which precisely the reverse of these conditions may be

* Address at the Meeting of the Association of Medical Officers of Asylums for the Insane, delivered at Edinburgh, July 28, 1858. See article "Lunacy Legislation," p. 523 of the present vol.

found, and it is against establishments of that class that security is desired. Need we say that the only real security would lie in their total abolition ? . . . . With regard to public asylums we have nothing to observe, for there can be no doubt that it is not in institutions of that class that the great abuses occur." Had the writer referred to a few of the later Reports of the Lunacy Commissioners, he would have ascertained how essentially erroneous the last remark is; and if we were to apply his arguments to public asylums, the result would be that we ought to agitate for their abolition also! No benefit is to be obtained from this style of reasoning, and improvement is retarded by having recourse to it, the attention being diverted from the true questions at issue. The *Times* does not, however, overlook the insuperable objections to any such sweeping reform as that which it advocates; for it remarks that "it is impossible to resist the conclusion that the public will have asylums of this kind in one form or other."

It is necessary that this very natural feeling of the public should be respected; and it would be well if the press, while advocating a stricter attention to the civil rights of the insane, would also give some little thought to their social rights. The law in its present state, while aiming at a due care of the civil rights of the lunatic, ingeniously throws most formidable obstacles into the way of many individuals, suffering from incipient mental affection, receiving that care which would be given to an individual suffering from any other malady. The person who has manifested the earlier symptoms of a disordered mind is often altogether debarred from receiving proper medical attention, unless he be first certified to be and registered as a lunatic—as being, in short, that which the medical man is anxious to prevent him becoming! As a medical question this is a matter of the greatest importance, and as a social question it is equally important; for the law often renders it requisite for individuals to be registered as lunatics, to their serious social detriment, who are suffering from a temporary and readily curable disturbance of the functions of the brain (see p. 532). Until the press and the public will regard this question respecting the medical attention of persons suffering from incipient symptoms of lunacy in its proper light, we cannot hope for a comprehensive improvement of the legal and general care of lunatics, and for a diminution of the false notions which are still prevalent respecting asylums.

The following remarks from the *Daily News* are worthy of being quoted:—

"A sweeping reform in the whole system of treatment of lunatics is urgently called for. The paltry palliations suggested are utterly worthless. It is proposed to abolish private and tolerate none but public asylums. But if the socalled public asylums are to be under the management of the same men who conduct the private asylums, what will be gained by the change? The truth is, almost every improvement that has been made in the treatment of the insane of late years has originated in private asylums. This is natural: the keeper of the private asylum depends for success upon his reputation; the keeper of a public asylum has no such stimulus to improvement. What is wanted is, that the Legislature, taking advantage of the lights of modern discovery, should clearly define the rights of individuals, and limit the physician's power of coercion. Within these limits the skill and humanity of the physician ought to be liberally trusted. But an efficient machinery should be organized to see that the law is respected, and to enforce it if need be. At present the law is vague and the machinery defective. There is no adequate security for any person alleged to be mad. There is no adequate security for the public against real madmen. The first thing to be reformed is the Commission of Lunacy; the next is the law which the Commissioners are supposed to enforce."

It is not often that a suicide occurs of so strange a character as that recorded in the following paragraph. Is the recital a fact, or an extravagant fiction?

"There is a furnished hotel in the Quartier St. Denis," says the *Droit*, "which is principally occupied by junior clerks. There is a large room in common for them, where those who happen to be without employment pass their time in playing cards or talking. The day before yesterday one of them, named Emile D——, said to his companions in a jocular way that it was so hot, and he was so out of spirits, that he had a strong inclination to blow his brains out. One of the young men present said he would make a bet against his doing such a thing. 'What will you bet?' replied Emile, still in the same laughing tone. 'A bottle of beer.' 'Done,' said the other, 'but order the beer at once, for as, to gain the wager, I must shoot myself, I should like to drink my share of it first.' The beer was ordered and drunk, when Emile rose up to leave the room. 'Where are you going?' said the others. 'To shoot myself,' was the reply, which was received with a burst of laughter from all present. Their merriment was, however, immediately put an end to by the report of a pistol in an adjoining room, and on running to the spot they found the young man lying dead on the floor. As no clue to his family could be found, the body was conveyed to the Morgue."—*Times*.

The *Saturday Review* has the following excellent remarks on this case:—

"The real stimulant to suicide is not reflection, but that absence of reflection which is engendered and fostered by society—by the constant intercourse, that is, of men and women bound together in some other way than by the ties of family life. The two greatest conquests of man, the two richest fruits of his wisdom, his experience, and his cultivation, are the virtue of women and the respect for human life. It is only by infinite pains they can be established, and it is only by infinite pains they can be preserved. For the society which profits by them in order to gain refinement and stability, threatens them with the very refinement and stability of which they are the most efficacious causes. As men get knit together, as life becomes more complex, as the surface of things is more polished, everything seems safe, and we transfer the security we feel from this world to the next. A sort of half belief steals through the mind that the scheme of divine justice has been arranged more pleasantly than it used to be, and it is hoped that God has withdrawn himself except from the casual purposes of a spasmodic mercy. Under the influence of these feelings, society begins rapidly to decompose. The virtue of women is treated as the dream of boys, and life is valued at a pot of beer.

"The novelists of modern France have represented this phase of human action in every possible light, and if they were charged with not drawing from the life, this foolish lad and his wager would justify them. The savages of a Pacific island could scarcely make lighter of chastity and existence than the inhabitants, as painted by the novelists, of the first continental city of civilized and Christian Europe. And the two things are always connected. The heroine goes through the easy process which scarcely deserves to be called falling, because her lover is going to shoot himself, or has even gone so far as to shoot himself partially. The hero, on the other hand, cocks his pistol at every turn of the intrigue. If the lady is momentarily stern, he has a good cry, and looks to his priming; if she yields, he feels his destiny is accomplished, and begins to trifle with the trigger. Nothing but the nicest art and the extremest finish of Parisian coquetry can keep the poor creature alive till the last chapter, and then, if he kills himself, he may die like a dog, and nobody cares. So pervading are these thoughts, that French novels are apt to be constructed on a pattern monotonously the same. But truth is often stranger than fiction, and art should gather resources from every quarter. What a *coup* it would be if some writer of romance were to take a hint from this newspaper story, and introduce a love-scene of appropriate passion and violence between the pot of beer and the explosion of the pistol!

"Suicide from recklessness of life is but the last stage of a descent along which the intercourse of society is apt to hurry all who give themselves up to it. The tendency to feel safe is almost irresistible under the excitement of life in a large city, and with this sense of safety come temptations that appear scarcely possible when the feeling of responsibility is again awakened. If this poor wretch could have had ten minutes alone in a field, he would probably have gathered strength to forego his beer. The sovereign preservative against this influence of society is family life. Its sorrows and its joys alike check the fever of the soul brought on by reckless security. But unfortunately this remedy is not, and cannot be, as universal as it is powerful. There are many persons for whom family life is a practical impossibility. But they have an antidote still left, and this is solitude. They can sometimes be alone, absolutely and consciously alone. This will be the salt of their lives, and solitude will restore them to themselves. If, alas! the salt has lost its savour, wherewith shall it be salted?"

In July suicides became so numerous in London that one of the weekly journals devoted a column, under the significant heading of "The Suicide Mania," to the records of such cases as had occurred during the seven days. Whether there was an excess of suicides in that month as compared with the same month in previous years, or whether there was an excess during the quarter as compared with previous quarters and the same quarter in the preceding year, cannot be told until the mortality records for the quarter are complete.

Several curious instances of suicide committed by young persons have recently occurred. A lad of twelve years of age, who had been convicted of some trifling offence and sentenced to twenty-one days' confinement in Chester Castle, hung himself in his cell. The circumstances of this case are not well known, but from the mother's statement it would seem as if there had been some unnecessary harshness in the punishment.

The following is an account of an inquest held upon the body of a boy who had committed suicide in Camberwell:—

"Yesterday (July 6th) Mr. W. Carter held an inquiry at the Kentish Drovers Tavern, Camberwell, respecting the somewhat extraordinary death of a boy, named John Thomas Cousens, aged thirteen years, who destroyed himself under the following circumstances:—

"Samuel Fry, having been sworn, said that he lived with his parents at No. 11, Sumner Street, Commercial Road, Peckham. The deceased resided next door. On Friday last he (witness) was sent by his mother on an errand, and when near the canal bank at Peckham met deceased, and he (witness) then told him that his father was looking after him, as he had run away from home. The deceased said, 'I don't care if I did stop away, as father said he should not take the trouble to come after me.' On returning towards home, the deceased said to him as they were walking by the canal bank that he 'liked being near the water,' and shortly after making this remark he went into the water, just over his boots, and came out again; and then, after remarking that they (meaning his parents) would never see him again, rushed into the water, and, after struggling about, sank. An alarm, however, was given, and a young man named Robert Hastings having procured the drags, with assistance the body, after about sixteen minutes, was taken from the water.

"It further appeared from the evidence of Thomas Cousens, deceased's father, and Ann Cousens, his mother, that the deceased left home on Thursday to go to work, but after he had been gone about an hour it was found that some money was missing; and, as the deceased had been guilty of stealing before, it was believed he had taken it. The deceased stayed out all night, and was never seen alive again by the parents. The father was present when the body was taken from the water. The body was conveyed to the Kentish Drovers, where every means were taken to restore suspended animation, but without effect.

"It further appeared that the deceased had before left his home, staying out all night, and had been corrected by his father on his return.

"Some other witnesses having been examined, the court was cleared of strangers, and the jury in about an hour returned the following verdict:—'The jury consider that the deceased no doubt went into the water for the purpose of destroying his life, but there was not sufficient evidence to show in what state of mind he was at the time; and they likewise believe that he was afraid to go home for fear of correction.'

"The inquiry then terminated, having lasted some hours."—*Standard.*

Here is another instance:—

"On Friday (September 10th), Mr. W. H. Phillips, deputy coroner, opened an inquest at the Fighting Cocks Inn, at Blakenall, near Wolverhampton, on the body of a young girl, Emma Griffiths, who committed suicide under the following distressing circumstances. William Cartwright, a blast furnace man, deposed that at about eleven o'clock on Wednesday morning he saw the deceased running along the road leading from Penn to Bilstone. Her father was about twenty yards behind. When she came near to Dainty's garden, she turned along a by-road leading to the Cock Street engine, ran up to an old pit which is about six feet from the path and twenty yards from the turnpike-road, and got over the wooden hoarding placed round the shaft. Witness was quite sure the deceased threw herself down the shaft wilfully. The scaffolding upon which she fell was sixty-nine yards deep. Joseph Elwell, engine-man, stated that he was let down by a rope into the pit. He went down three times, and dragged for her through thirty feet of foul air. At last he got air-troughs, which cleared the shaft to within nine feet of the scaffolding, and he then recovered the body. John Griffiths, father of the deceased, said she was fifteen years and four months old, and had for about six months been living in service at William Traunter's, a beershop-keeper close by. She came home on Sunday morning, and said her master had been 'pulling her about.' Witness replied, 'Then, my wench, I'll make an example of him.' She was very much agitated, and then and on Monday and Tuesday the sight of a man seemed to frighten her. Witness on Tuesday instructed her to meet him at the magistrate's clerk's office. She failed to do so, and when he went home at night he found her in bed. He asked her why she did not meet him. She said, 'Father, I was ashamed; I am very bad.' He said he should take her to a surgeon's; but on Wednesday morning, he found upon getting up that she had left home. He traced her to the Penn turnpike, and, accompanied by his little boy, about five years old, followed her slowly at a distance of about thirty or forty yards. When she got near the Fighting Cocks she ran towards a pit. Witness then shouted out to her, 'Oh, my wench, what are you going to do?' She did not, however, heed him, and rolled herself over the fencing around the pit into the shaft. He did not believe that she knew what she was about; for, on the Sunday, when Traunter came into the yard, the deceased became dreadfully agitated, and wanted to get out at the front window. The coroner inquired whether on any previous occasion the deceased had complained of ill-treatment. Witness said she had not, but when he questioned her respecting Traunter's previous conduct, she said that he had 'pulled her about before,' but he had never 'served her so bad as on Sunday morning.' The jury expressed themselves in strong terms upon the alleged conduct of Traunter. The father said he hoped this man would be punished. The coroner replied that, if all was true that had been stated, Traunter richly deserved punishment. He, however, had no power to interfere, but recommended Griffiths to consult a solicitor. The father said he was a poor man, and did not know how he could find the means to institute a prosecution. After some further evidence had been taken, the inquest was adjourned until Wednesday next, to give time for a *post-mortem* examination."—*Times.*

These three cases derive their principal interest from the age of the individuals.

Among comparatively recent suicides is recorded that of Henry William Herbert, a popular writer, better known as "Frank Forrester." He shot himself, when in company with a friend, at a lodging-house in Newark, U.S., and it is stated that domestic difficulties led to the commission of the rash act. He had been married about three months previously, but he had not lived with his wife more than six weeks when they quarrelled, and she separated from him. Herbert suffered from intense mental agony in consequence of the severe blow, and in his ravings often threatened to commit suicide. After the act had been committed, a letter, of which the following is a copy, was found in his writing-desk, addressed to the coroner:—

"Tuesday, March 18th, 1858—(Three months since the happiest day of my life)—To avoid all trouble and simplify your duty, I have to state that I have taken my own life with a pistol, no one being privy to my doing so, or to my design. My reason for this act consists in no remorse for anything that I have done or left undone—from no pecuniary pressure—from no inability or fear of inability to support myself—from no weak fear of public opinion, least of all of the public opinion of Newark, which I do now, as I have always done, utterly disregard and despise—from no embarrassment arising from any indebtedness. I have abundance of employment, and the prospect of much more. Had the people of Newark, whom I forgive from the bottom of my heart, suffered me to live harmlessly and happily in my humble home, and to amend my life where it was in error in a new sphere, which I was honestly prepared to do, I might have paid off all my debts and lived many years among you an honest, useful, and happy man. My debts will be paid from assets to the last dollar. It was not, however, so to be. My blood and the guilt of it are upon those men and women of Newark who first sowed suspicion, distrust, and dissension between myself and the sweetest creature God ever gave and man took away from an unhappy sinner. My own unhappy temper did the rest. The reason for this act is simple. My life, long sad, and solitary, and weary, and without an object beyond labour to earn a living for the day, has become utterly hopeless, hateful, and unendurable. A hope had been kindled in my heart; again my home had got a light brighter than sunshine—my life had a purpose; I loved her unutterably—happy; all this has been dashed down, all is lost for ever—home, hope, sunshine. She let life go lifewise; since henceforth it is another word for torture. I would not deny falsely one fault of which I am conscious, especially at this last moment; I would not deny that I erred towards her whom this day shows I loved more than life. I did err, but it was hastily, in rash act or rash word—never, so may God deal with me, in thought or intimation. I never had a word with her about money matters, nor cared nor scarcely knew whether she had or had not money. I never laid a hand or finger on her in wrath in my life. What I did or said wrongfully, I repented on the instant. I have endeavoured to atone for it ever since. I die for it this day. I think, I hope I deserve pity more than blame; but I know that I shall not find it, least of all in Newark. I can say truly with my last breath I never wronged a man or woman in my life by premeditation, or failed to ask pardon and make atonement when I could do so. I never bore malice in my life. I repent of all my faults and sins, and have endeavoured to amend them. I die in perfect peace and charity with all men; I beg forgiveness of all those against whom I have sinned; I forgive all those who have sinned against me, even the woman who called at my own house, and set my wife's thoughts first against me—in proof of it I am sure I know her, but do not name her name; I beg God to forgive me, as I forgive all my enemies. I die in perfect faith and trust in my Redeemer, and believe that in Him I shall have eternal life.

"Henry William Herbert." *

What a painful picture of capricious irritability and moroseness, verging on insanity, have we here! Does not every portion of this statement point to the existence of some obscure disease of the brain? Had the exacerbations of temper of the unfortunate man (singularly irrational as they must have been, as may be gathered from the record before us,) been looked upon as the result of an abnormal condition of the brain, is it not probable that his life might have been saved, and his domestic comfort secured, by the aid of a kind and sympathizing physician?

Whether the imprisonment of the lad who committed suicide in Chester Castle be considered an act of thoughtless severity or not, incidents are unfortunately not wanting to show that the reformatory movement is not so well appreciated on the bench as it ought to be, and that too many of our magistrates still adhere to the vicious old dogmas of punishment. For example:—

* *New York Herald.*

"At the Rochdale Petty Sessions, on Thursday, Owen Glancey, an Irish boy, aged twelve years, was charged with robbing the garden of James Cheetham, of Foxhole's Lane, on Tuesday. Mr. Cheetham caught him in the garden, and on searching him found upon him eight gooseberries! It was stated that the prisoner had done a similar thing before, and the bench sentenced him to prison for one month with hard labour."—*Daily Telegraph*, Aug. 9th.

Again, in the House of Lords (June 28th):—

"Lord Brougham called the attention of the House to the case of a boy only eight years of age, who, in the absence of his parents and of any person connected with him, and in the absence, moreover, of all legal evidence, had been sentenced by some judicial tribunal in Edinburgh to be imprisoned and flogged. That sentence had actually been carried into effect, and the poor boy went forth into the world branded as one who had been flogged in the most ignominious manner. Since then the conviction had been quashed. He had received a letter from a professional gentleman, who had interested himself in the case, stating that upon an action being brought a large sum was obtained as compensation for the boy, and his friends thought that under the circumstances—the conviction being quashed—his character was sufficiently vindicated. The boy's friends might be satisfied, but he should not be satisfied, nor would justice be satisfied, without further inquiry. (Hear.) He hoped there would be no further delay in the production of the paper for which he had moved."—*Times*.

Such perversions of justice as these examples show are simply monstrous; it is a reproach to the whole of the bench in the neighbourhood where they happened that they were not repudiated.

Among the criminal events of the quarter, the horrible murder at Darley, in Yorkshire, holds—not only from the terrible character of the deed, but also from the singular psychological study it affords—the most prominent place; but as the whole circumstances of the case (notwithstanding the criminal's confession) cannot be fully entered into until after the trial of the murderer, we shall postpone any consideration of it until our next quarterly retrospect.

